# Expression Profiling and Functional Characterization of miR-26a and miR-130a in Regulating Zhongwei Goat Hair Development via the TGF-β/SMAD Pathway

**DOI:** 10.3390/ijms21145076

**Published:** 2020-07-18

**Authors:** Yangyang Ding, Xianglan Xue, Zhanfa Liu, Yong Ye, Ping Xiao, Yabin Pu, Weijun Guan, Joram Mwashigadi Mwacharo, Yuehui Ma, Qianjun Zhao

**Affiliations:** 1 Key Laboratory of Animal (Poultry) Genetics Breeding and Reproduction, Ministry of Agriculture and Rural Affairs, Institute of Animal Sciences, Chinese Academy of Agricultural Sciences (CAAS), Beijing 100193, China; dyy563078081@163.com (Y.D.); larrykazunari@126.com (X.X.); puyabin@caas.cn (Y.P.); guanweijun@caas.cn (W.G.); 2 The Ningxia Hui Autonomous Region Breeding Ground of Zhongwei Goat, Zhongwei 755000, China; xqycbj@126.com (Z.L.); larrykazunari@gmail.com (Y.Y.); 3 Farm Animal Genetic Resources Exploration and Innovation Key Laboratory of Sichuan Province, College of Animal Science and Technology, Sichuan Agricultural University, Chengdu 625014, China; pinshaw@163.com; 4 International Center for Agricultural Research in the Dry Areas, Addis Ababa 1000, Ethiopia; j.mwacharo@cgiar.org; 5 CAAS-ILRI Joint Laboratory on Livestock and Forage Genetic Resources, Institute of Animal Science, Chinese Academy of Agricultural Sciences (CAAS), Beijing 100193, China

**Keywords:** Zhongwei goat, microRNA, curly hair, papilla cells, *TGF-β/SMAD*

## Abstract

The Zhongwei goat is an important and unique goat breed indigenous to China. It has a natural hair curling phenotype at birth, but the degree of curling gradually decreases with growth. The molecular mechanism underlying the dynamic changes in the wool curvature in Zhongwei goats is poorly understood. MicroRNAs (miRNAs) play important roles in many biological processes, including hair growth and development. In this study, we selected skins from Zhongwei goats at different ages (45 and 108 days) that exhibited different levels of hair curvature and performed miRNA sequencing to explore the molecular mechanism of hair bending. In total, 28 significantly differentially expressed miRNAs (DE miRNAs) were identified in the three groups of samples between the two developmental stages. An analysis of the target genes of the above-mentioned DE miRNAs by the Gene Ontology (GO) and Kyoto Encyclopedia of Genes and Genomes (KEGG) pathway analyses indicated that the DE miRNAs were involved in signal pathways which were previously associated with hair bending and hair follicle development, such as the *TGF-β/SMAD*, *PI3K-Akt*, *JAK-STAT*, and *MAPK* pathways. A comprehensive analysis of the correlations between the miRNA-seq results and issued transcriptional findings indicated that *SMAD1* was a target gene of miR-26a and *SMAD5* was a target gene of miR-130a. Furthermore, goat dermal papilla cells were successfully isolated and purified to determine the role of miRNAs in follicle development in vitro. The study results demonstrated that miR-130a and miR-26a had significant effects on the proliferation of dermal papilla cells. In addition, the detection results of mRNA and protein levels indicate that the overexpression of miR-26a can promote the expression of related genes in the *TGF-β/SMAD* pathway, while miR-130a has the opposite substitution effect. The dual luciferase report test showed that miR-26a targeted the *SMAD1* gene and reduced the expression of the *SMAD1* protein in hair papillary cells. Our results identified DE microRNAs which perhaps change at the time of hair straightening in Zhongwei goats and explore the role of miR-26a and miR-130a in dermal papilla cells proliferation. The present study provided a theoretical basis to explore the mechanisms underlying the Zhongwei hair growth and curly phenotype.

## 1. Introduction

The Zhongwei goat is a famous native Chinese goat breed used for high quality suede. The principal production area of the Zhongwei goat is located in Xiangshan in Zhongwei City, Ningxia Hui Autonomous Region. The wool of a Zhongwei one-month lamb is approximately 7 cm long with highly curved patterns. However, from two to three months after birth, the delicate flower pattern disappears gradually due to a reduction in the bending of the hair strands and the looseness of the wool strands, which remarkably affects the economic value of the coat. However, the molecular mechanism underlying this short-term change in hair shape is still unknown.

Auber suggested that the bending direction of the hair ball structure at the bottom of the wool follicles is opposite to that of the hair structures at the top [1]. For uniformity and curvature characteristics, wool bending may be a feature that is determined during embryonic development. This is mainly due to the consistency of the depth of fetal hair follicles in the skin and whether the growth direction is uniform among diverse populations that can be attributed to the asymmetric [2]. Previous studies have revealed that hair bending is caused by an irregular growth and bending of the hair follicles in the epidermis, the asymmetric distribution of proteins producing different biological forces, which squeeze the hair into a variety of shapes [3].

The regulatory mechanism underlying the hair curl trait formation is still elusive [4,5,6,7]. The molecular mechanisms underlying hair follicle progression, growth and hair curl are similar in mammals [8]. These research results provide a theoretical basis to illuminate the molecular mechanism underlying wool curl formation. In recent years, the study of hair fiber curl formation using mice as a model has accelerated our understanding on the genetic mechanism underlying hair curl traits. Classic signal pathways, such as the *Wnt*, *EDA/EDAR*, *TGF-β/SMAD*, *BMP*, *EDA/NF-κB*, and *Shh* pathways, have been found to be related to hair bending [9,10,11,12]. Andl et al. reported that Dicer and miRNAs are expressed in mouse skin tissue, suggesting that miRNAs play major roles in the development of skin and hair follicles [13]. Previous studies have shown that miRNAs have two characteristics in regulating gene expression: temporal specificity and tissue specificity, which determine the direction of cell differentiation [14]. Similarly, during the growth of the epidermis and hair follicles, the miRNA expression profile also exhibits these properties, i.e., some miRNAs are only expressed during a specific period or in a specific tissue. For example, the expression level of miR-184 gradually increases during the growth period of sheep hair follicles, and the expression level of miR-205 increases and then decreases from anagen to telogen, with the highest expression in catagen [15]. Kang et al. screened candidate genes determining wool growth and functional clusters closely related to this process in Chinese Tan sheep at 1 month after birth (curly wool) and 48 months after birth (straight wool) by transcriptase sequencing [16]. These results shed light on our experimental design. Nissimov et al. [17] proposed the hypothesis of multiple dermal papillary (MPC) transitions in which hair follicle growth is divided by the base of the hair bulb. It was believed that hair bending was caused by a partial separation of the dermal papilla at the bottom of the hair follicle. This phenomenon produces multiple hair fibers that are wrapped around the stratum corneum with different discrete hair shaft tips as the axis. In addition, this phenomenon is related to the degree of separation of hair papilla cells; the more independent the structure of each subunit is, the greater the difference in the predetermined center, which gives the subunits different growth speeds, resulting in the generation of bending. Therefore, we chose caprine dermal papilla cells as the model to explore miRNAs function in hair follicle development in vitro.

In recent years, there have been several studies related to wool shape and wool quality identification [18,19,20]; however, these studies mainly focused on genetic differences among different populations with divergent morphological wool types. Few studies have concentrated on the molecular mechanism underlying the dynamic changes of wool morphology at different development stages. Here, we identified the expression profile of Zhongwei goats at two different ages (45 and 108 days) by high-throughput sequencing. To construct a network related to hair development, we analyzed and tested potential miRNA/mRNA interactions. We sought to investigate the roles of miR-26a and miR-130a in hair development. The aim of this study was to provide a theoretical basis for the identification of the potential miRNAs involved in the wool growth of Zhongwei goats and to further discover the regulatory mechanism underlying the hair follicle development in animals.

## 2. Results

### 2.1. Identification of miRNAs in Goat Hair Follicles

**Figure 1:**
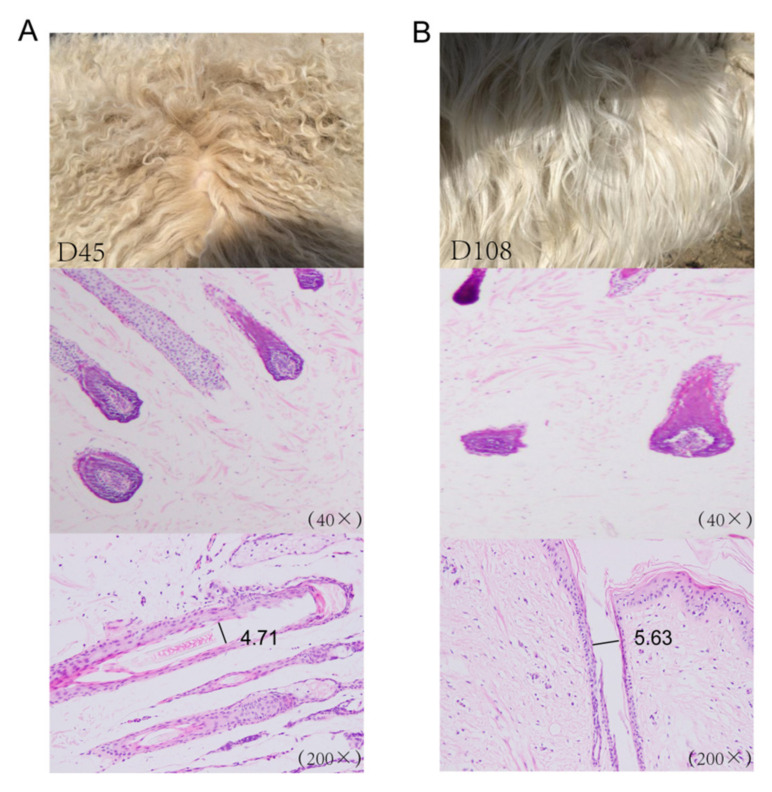
The dynamics of hair curling. (**A**) The pelt of a Zhongwei goat at 45 days old and a section of a single hair follicle. (**B**) The pelt of a Zhongwei goat at 108 days old and a section of single hair follicle.

To investigate the mechanisms underlying wool fiber development in two postnatal stages (45 days and 108 days), we sampled scapular skin tissues from three unrelated Chinese Zhongwei goats at two postnatal stages (45 days and 108 days). We examined the internal structure of the skin and hair follicles in the scapular region of the Zhongwei goats in the skin samples harvested at the two time points. Compared with that at 45 days, the depth of the hair follicles at 108 days was shallower, and the hair bulbs were wider. Additionally, the cortex became thinner and the diameter became wider (Figure 1A,B).

**Figure 2:**
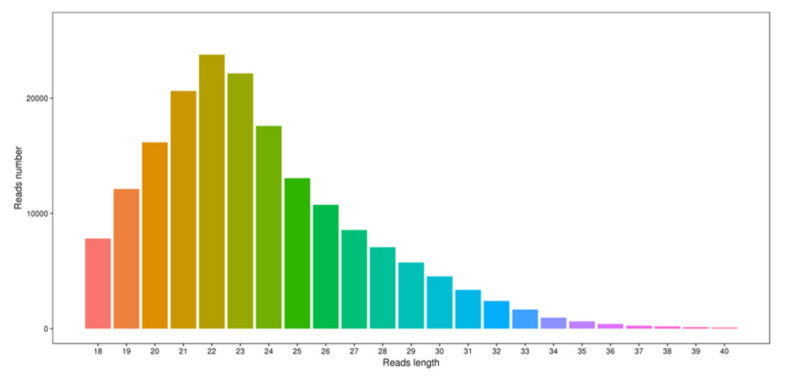
Small RNA length analysis statistics.

To study the changes in the miRNA expression associated with the different wool phenotypes exhibited in Zhongwei goats at different developmental stages, we performed a sequencing of six libraries representing scapular skin tissue samples obtained from three animals at 45 days and 108 days using an ILLUMINA HiSeq 2000 platform. We obtained 1351.68 Mb, 805.19 Mb, 866.27 Mb, 1290.24 Mb, 1530.00 Mb, and 1280.00 Mb amount of raw data from the six samples, respectively. After the preliminary quality control filtering, an average of approximately 73% clean data were obtained (Supplementary Materials Table S1). We also performed a statistical analysis of the length distribution of high quality sRNA reads (Figure 2), and the results showed that the length of the total reads was primarily 22 nt, which accounted for 30.56% of the reads, and that the proportions of reads that were 20, 21, and 23 nt in length among the distinct reads were 4.24%, 12.31% and 13.46%, respectively.

### 2.2. Analysis and Validation of DE miRNAs

**Figure 3:**
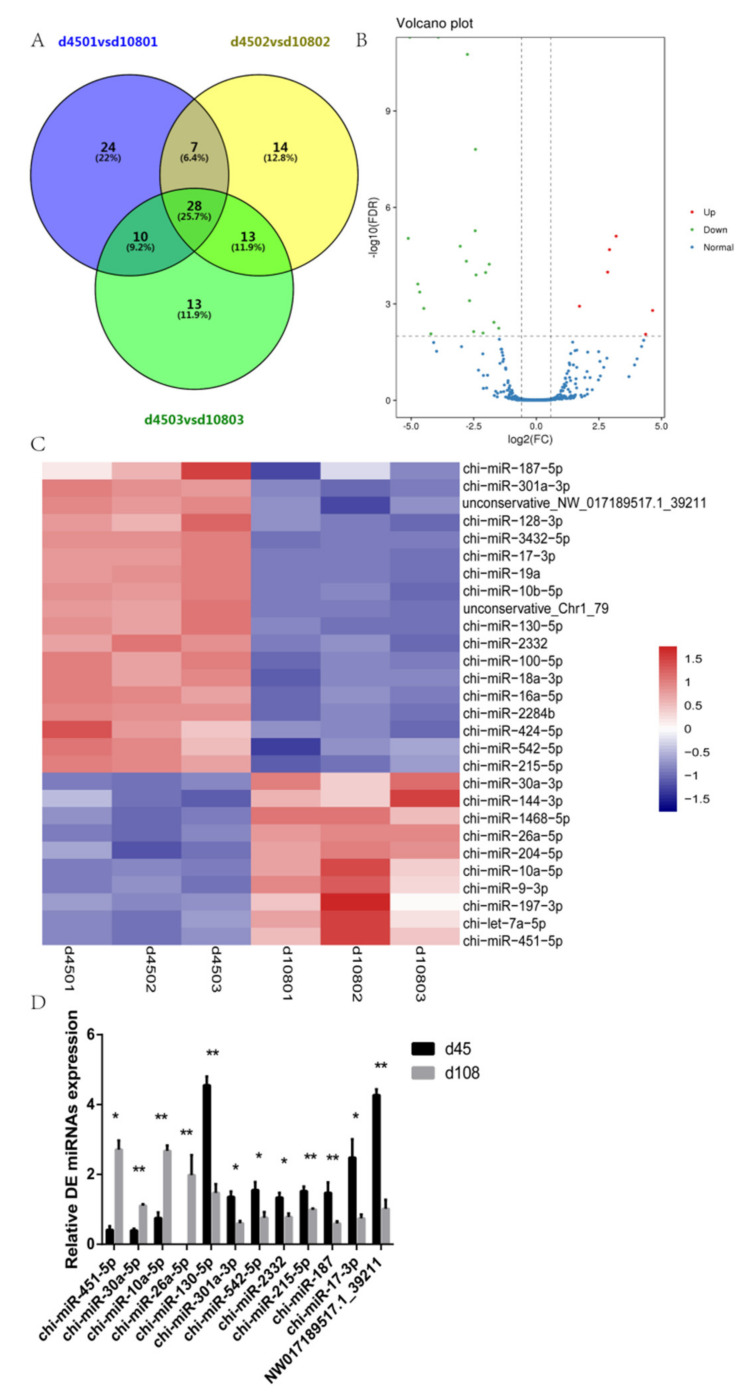
The miRNA-seq results analysis and validation. (**A**) A Venn diagram of differentially expressed miRNAs (DE miRNAs), d4501 vs. d10801 represents the number of differential miRNAs of the first sheep in two different periods, d4502 vs. d10802 and d4503 vs. d10803 have the same meaning. The overlapping circles represent the number of differential miRNAs between the combinations. (**B**) A volcano map of differentially expressed miRNAs. The volcano plot shows the levels of expression of the DE miRNAs shared between the 45- and 108-day-old samples. (**C**) A heat map of miRNAs. The abscissa represents the sample name and the clustering result for the sample, and the ordinate represents the clustering result for the differentially expressed miRNAs. Red indicates upregulation, and blue indicates downregulation. (**D**) RT-qPCR analysis of twelve DE miRNAs, with the relative expression calculated by the 2^−ΔΔCt^ method; * *p* < 0.05 and ** *p* < 0.01.

We used the DESeq2 package to compare the miRNA expression between the two groups of each lamb samples. To control the false positive rate, the differentially expressed miRNA screening conditions were FDR < 0.01, and |fold change| > 1.5. DE miRNAs in each group, as listed in Table 1. In total, 28 miRNAs were differentially expressed in the three pairs of samples from the two time points (Figure 3A). Among them, 18 miRNAs were more abundant in the 45-day-old group than in the 108-day-old group, and 10 miRNAs were less abundant in the 45-day-old group than in the 108-day-old group (Figure 3B). We selected 12 of the 28 DE miRNAs as candidate miRNAs and performed a cluster analysis (Figure 3C). To verify the miRNA-seq results, the expression levels of 12 miRNAs were determined by RT-qPCR. The expression levels of chi-miR-2332, chi-miR-301a-3p, chi-miR-542-5p, chi-miR-130-5p (miR-130a), chi-miR-215-5p, chi-miR-187-5p, chi-miR-17-3p and unconservative_NW_017189517.1_39211 in 45-day-old lambs were significantly higher than those in 108-day-old lambs. In contrast, the expression levels of chi-miR-451-5p, chi-miR-30a-5p, chi-miR-10a-5p and chi-miR-26a-5p (miR-26a) were lower in 45-day-old lambs than in 108-day-old lambs (Figure 3D). These RT-qPCR results were consistent with the results of the miRNA-seq data analysis, indicating that the miRNA-seq data are reproducible and reliable.

### 2.3. Prediction and Functional Annotation of the Target Genes of Identified miRNAs

**Figure 4:**
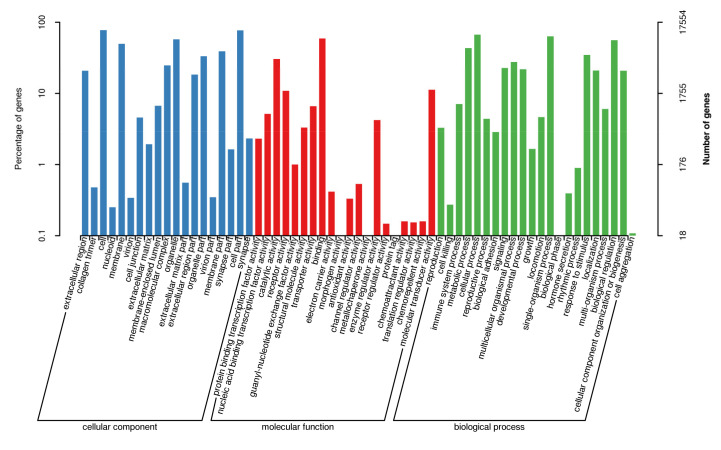
The Gene Ontology (GO) enrichment results. The abscissa is the GO classification, the left side of the ordinate is the percentage of the number of miRNA target genes, and the right side is the number of miRNA target genes.

**Figure 5:**
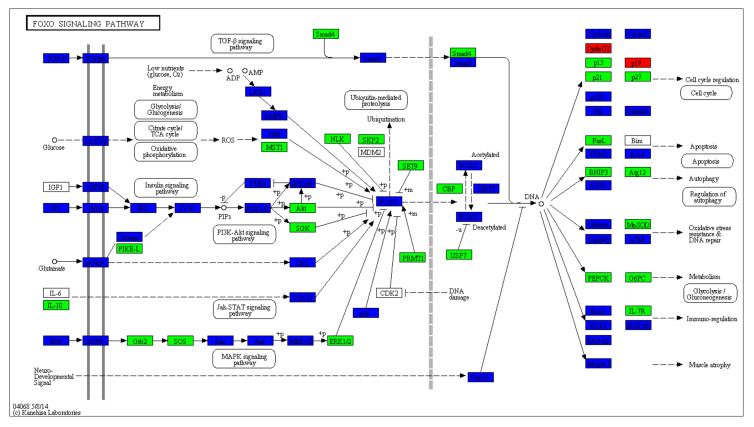
Annotated diagram of the Kyoto Encyclopedia of Genes and Genomes (KEGG) pathway analysis of differentially expressed miRNA target genes. The green-box-labelled enzymes indicate a downregulation of a miRNA target gene, and the blue-box-labelled enzymes indicate an upregulation of a miRNA target gene compared with the normal expression of the target gene.

A total of 1079 miRNAs were identified, including 411 known miRNAs and 668 novel miRNAs. We utilized RNAhybrid and miRanda software to predict the miRNA target genes. The predicted target genes were classified by Gene Ontology (GO) enrichment and categorized into 61 GO entries (Figure 4). Among the GO terms, the most significant enrichment terms were the organelle, cellular process, single-organism process, catalytic activity, nucleic acid-binding transcription factor activity, and immune system process. The annotated analysis of the pathways, including the target genes of the DE miRNAs, further interpret the functions of the miRNA target genes (Figure 5). The pathway annotation results for the target genes of the DE miRNAs included the *TGF-β/SMAD* signal pathway, *PI3K-Akt* signal pathway, *JAK–STAT* signal pathway and *MAPK* signal pathways, which may be involved in hair bending regulation.

### 2.4. Integrated miRNA and mRNA Analysis Identified Target Genes Involved in Curly Wool Development

**Figure 6:**
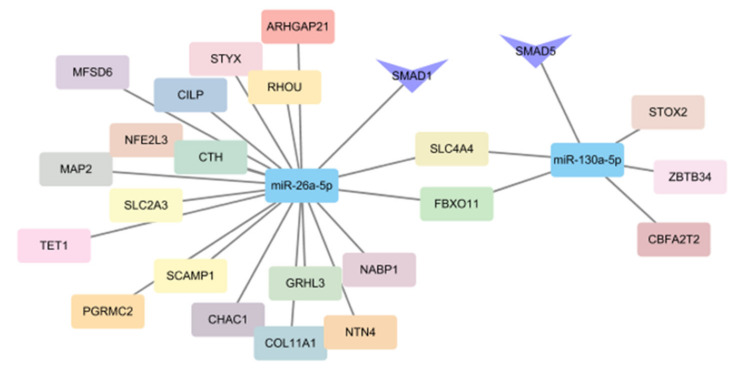
The miRNA−mRNA network associated with sheep wool curvature, showing the interactions among miR-26a, miR-130a and their target genes.

We selected two of the 12 candidate DE miRNAs that exhibited extremely significant differences and predicted their target genes. The predicted miRNA target genes were combined with published transcriptomic analysis data from our lab [21], to construct a mRNA- and miRNA network related to hair bending. Taking the intersection of the results obtained from the two prediction sites, we found that miR-26a has 667 target genes and miR-130a has 184 target genes. Then we intersected these results with 326 differential genes of transcriptome, and obtained a 20 and 6 gene overlap, respectively. Important mRNA−miRNA pairs that exhibited a reverse expression pattern between the 45-d and 108-d Zhongwei goat samples were selected. From the results displayed through Cytoscape 3.4.0., MiR-26a was predicted to have 20 coexpressed genes, miR-130a was predicted to have six coexpressed genes, and *SLC4A4* and *FBXO11* were common to these two miRNAs (Figure 6). Notably, in these coexpressed genes, both miR-130a and miR-26a were enriched in the *TGF-β/SMAD* signal pathway. These results were similar to the previous Kyoto Encyclopedia of Genes and Genomes (KEGG) pathway enrichment findings. Therefore, the *TGF-β/SMAD* signal pathway has a potential role in the formation of wool curvature and will be the focus of our follow-up work.

### 2.5. Isolation and Identification of Dermal Papilla Cells In Vitro

**Figure 7:**
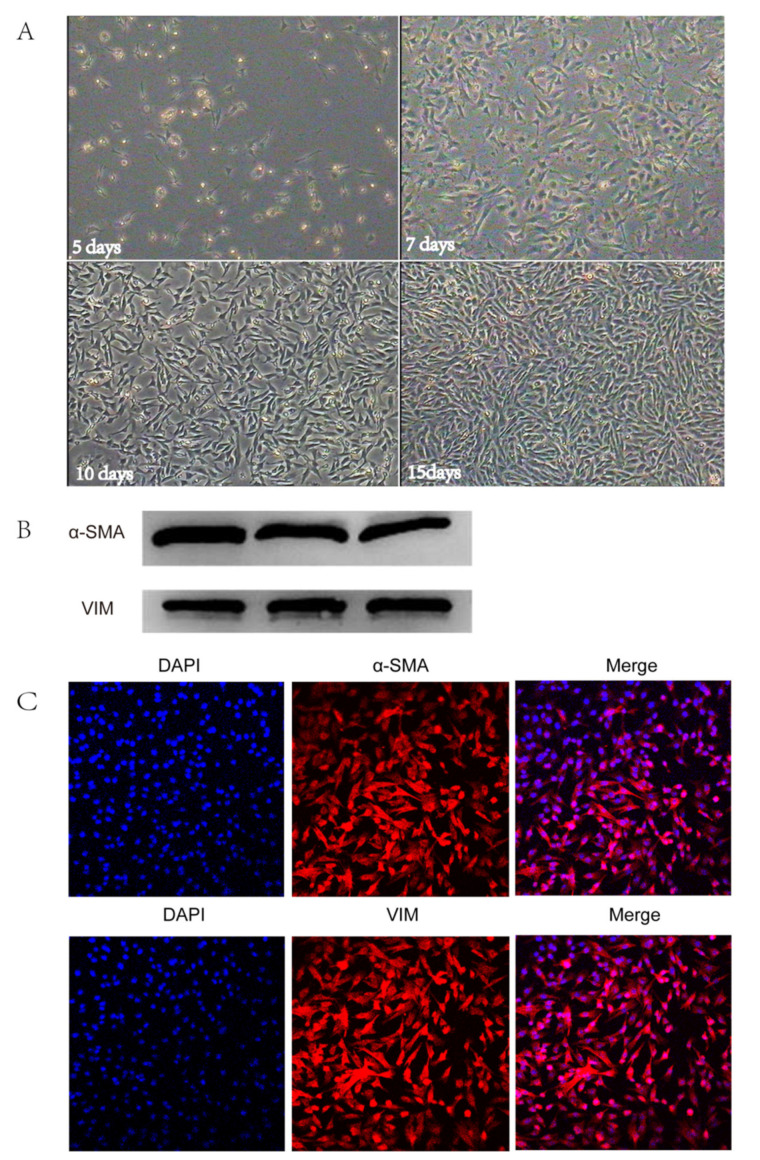
Isolation and identification of dermal papilla cells in vitro. (**A**) The in vitro-cultured dermal papilla cells show the growth of hair papilla cells for 5 days, 7 days, 10 days, and 15 days, respectively. (**B**) Western blotting for smooth muscle actin (*SMA*) and vimentin (*VIM*), with three repeats for each group. (**C**) Immunofluorescence staining of dermal papilla cells for α-smooth muscle actin (*α-SMA* (200×)) and *VIM* (200×).

To determine the role of miRNA in hair follicle development, we isolated dermal papilla cells from goat skins. Cell attachment was found in a 5-day cell-isolated culture. The proliferation rate of the cells was markedly increased at seven days. The appearance of the cells was triangular or polygonal, the cell body was large, and the cytoplasm was abundant. After 10 days of growth in culture, the dermal papilla cells began to increase radially, gradually losing their original shape and forming a dense region. The dermal papilla cells grew for 15 days before needing to be passed for the first time (Figure 7A). As the number of generations increased, the morphology of the dermal papilla cells gradually became fusiform, similar to that of fibroblasts. The difference is that the cell bodies were larger than those of the fibroblasts and grew in a spiral shape. The expression of the DP-specific marker genes, vimentin (*VIM*) and α-smooth muscle actin (*α-SMA*), was assessed in cultured DPSs. An anti-α-SMA antibody assay showed a distinct band near 43 kDa, which was consistent with the size of the α-SMA protein (42 kDa); an anti-VIM antibody assay showed a conspicuous band at approximately 55 kDa, consistent with the size of the VIM protein (56 KDa (Figure 7B)). In addition, immunofluorescence staining was also used to detect the expression of the specific marker (*α-SMA*) and the hair follicle dermal cell-derived marker (*VIM*) in cultured dermal papilla cells. The results showed that both markers were positive, as indicated by a red fluorescence, and the antibody expression was positive. DAPI nuclear staining confirmed that the expression of *α-SMA* and *VIM* were in the cytoplasm in DPCs (Figure 7C). These findings confirmed that the isolated cultured cells were the dermal papilla cells of the hair follicle.

### 2.6. Effects of miR-26a and miR-130a on Cell Proliferation

**Figure 8:**
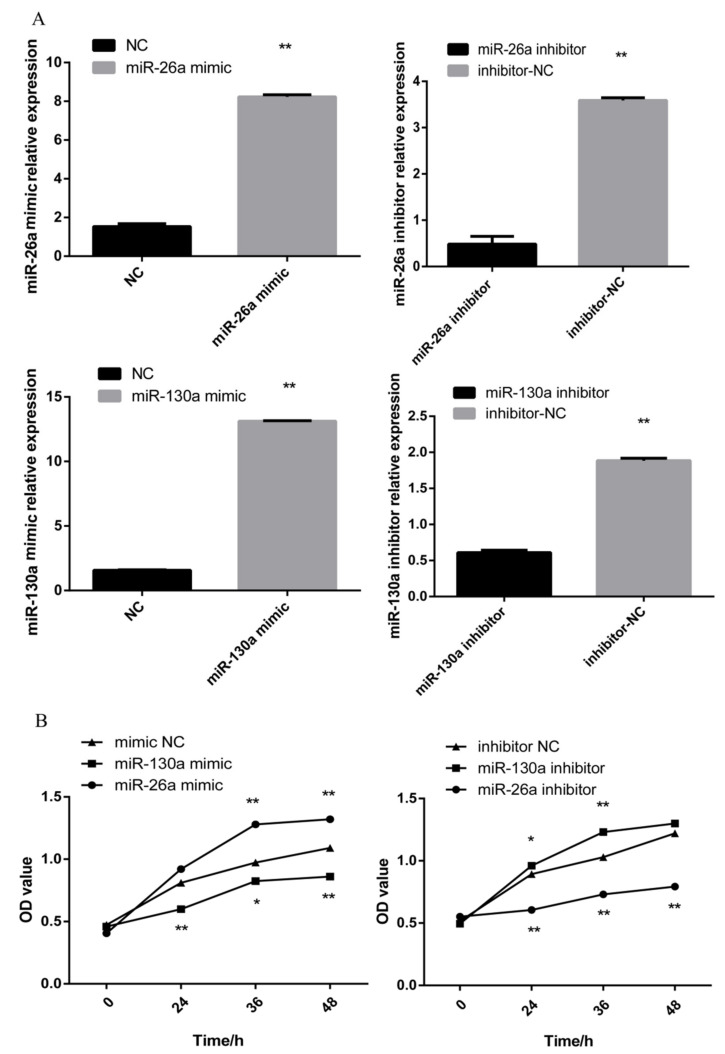
The proliferation of DPCs was prompted by miR-26a, and miR-130a inhibited the proliferation of DPCs. (**A**) The expression of miR-26a and miR-130a after a transfection with a miR-26a and miR-130a mimic, negative control (NC), and miR-26a and miR-130a inhibitor, was determined by RT-qPCR 48 h after transfection (*n* = 3). (**B**) Effects of the overexpression or inhibition of miR-26a/miR-130a evaluated by CCK-8 assays. The marker * represents a significant difference between the experimental group and the control group (*p* < 0.05), while ** represents an extremely significant difference between the experimental group and the control group (*p* < 0.01). All reactions of each sample were repeated three times.

After a transfection of 45 h, the overexpression and inhibition efficiency of miR-26a and miR-130a in DPCs were confirmed by RT-qPCR (Figure 8A). Then we tested the proliferation of the DPCs after the transfection of the mimics and the inhibitors of miR-26a and miR-130a by CCK-8 assays. The results showed that an overexpression of miR-26a significantly promoted the proliferation of DPCs and the inhibition of miR-26a suppressed the proliferation of DPCs. The proliferation of the miR-130a mimic-treated cells was significantly reduced, whereas that of the miR-130a inhibitor-treated cells was significantly increased (Figure 8B).

### 2.7. Effects of miR-26a and miR-130a on the TGF-β/SMAD Pathway

**Figure 9:**
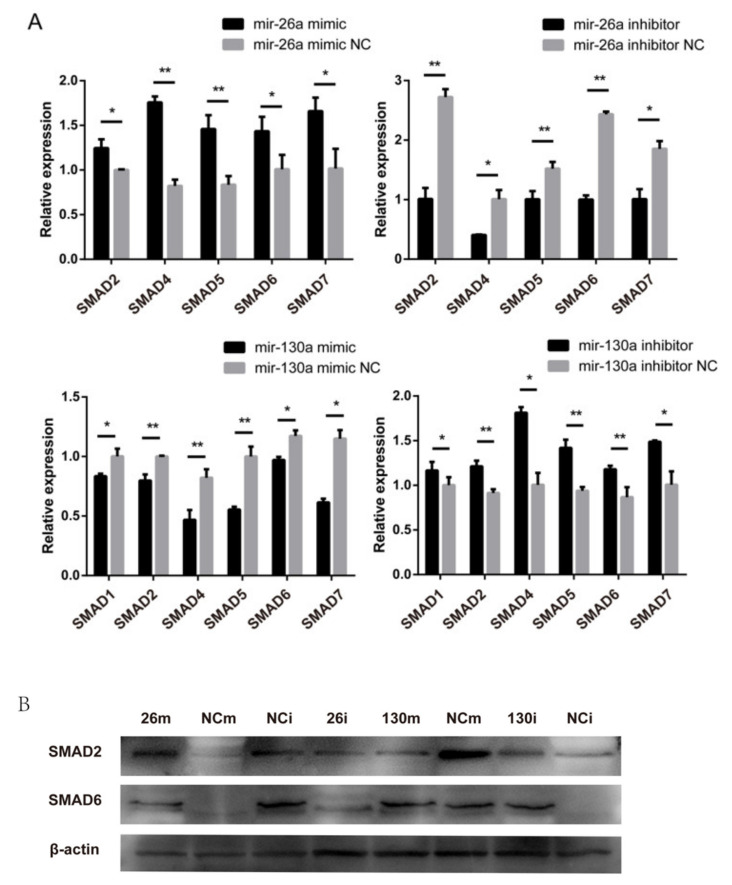
The expression of related genes in the *TGF-β/SMAD* pathway after the overexpression or inhibition of miR-26a or miR-130a. (**A**) The *SMAD* family expression after a transfection with a miR-26a and miR-130a mimic, negative control (NC), and miR-26a and miR-130a inhibitor, determined by RT-qPCR 48 h after transfection (*n* = 3). The marker * represents a significant difference between the experimental group and the control group (*p* < 0.05), while ** represents an extremely significant difference between the experimental group and the control group (*p* < 0.01). (**B**) Western blot analysis of *SMAD2* and *SMAD6* after a transfection with the miR-26a and miR-130a mimic, negative control (NC), and miR-26a and miR-130a inhibitor.

To validate the effect on the *TGF-β/SMAD* pathway, the expression levels of the major genes were determined after the overexpression/inhibition of miR-26a or miR-130a. First, we detected the expression of the related genes at the mRNA level by RTq-PCR. After overexpressing miR-26a, the expression levels of the *SMAD2*, *SMAD6* and *SMAD7* genes were significantly higher than those induced by the control treatment (*p* < 0.05), and the expression levels of *SMAD4* and *SMAD5* in the miR-26a mimic group were significantly higher than those in the control group (*p* < 0.01). Following the inhibition of endogenous miR-26a, the expression levels of the *SMAD4* and *SMAD7* genes were significantly lower than those induced by the control treatment (*p* < 0.05). The expression levels of *SMAD2*, *SMAD5* and *SMAD6* in the miR-26a inhibitor group were radically different from those in the control group (*p* < 0.01). Following the overexpression of miR-130a in dermal papilla cells, the expression levels of the *SMAD1*, *SMAD6* and *SMAD7* genes were significantly lower than those induced by control treatment (*p* < 0.05), and the expression levels of the *SMAD2*, *SMAD4*, and *SMAD5* genes in the miR-130a mimic group were significantly lower than those in the control group (*p* < 0.01). The expression levels of the *SMAD1*, *SMAD4* and *SMAD7* genes in dermal papilla cells in the miR-130a inhibitor group were significantly higher than those in cells in the control group (*p* < 0.05), and the expression levels of *SMAD2*, *SMAD5* and *SMAD6* were significantly higher in the inhibitor group than in the control group (*p* < 0.05 (Figure 9A)).

Next, we investigated the expression levels of the *SMAD2* and *SMAD6* genes in the *TGF-β/SMAD* pathway by western blotting. After the overexpression/inhibition of endogenous miR-26a or miR-130a in dermal papilla cells, the western blotting results were consistent with the mRNA expression level results (Figure 9B). The overexpression of miR-26a increased the *SMAD2* and *SMAD6* gene expression in dermal papilla cells, and the inhibition of miR-26a decreased *SMAD2* and *SMAD6* gene expression. *SMAD2* and *SMAD6* expression was also downregulated as miR-130a was overexpressed, while inhibiting miR-130a upregulated the expression levels of the *SMAD2* and *SMAD6* genes.

### 2.8. Dual Luciferase Reporter Gene Detection

**Figure 10:**
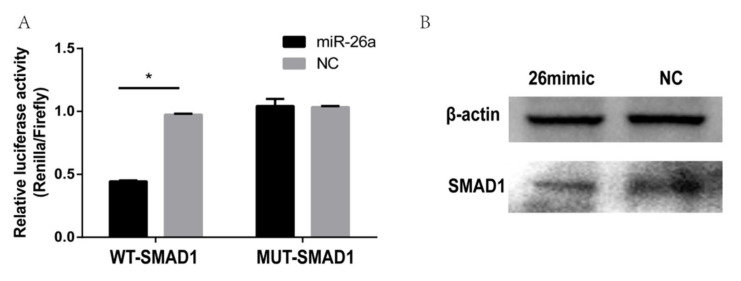
Verification results of the dual-luciferase reporter system of miR-26a and the target gene *SMAD1*. (**A**) Expression of relative luciferase activity in wild type vector (WT group) and mutant (MUT group). The marker * represents a significant difference between the experimental group and the control group (*p* < 0.05). (**B**) Western blot analysis of *SMAD1* after a transfection with the miR-26 mimic, negative control (NC).

To further investigate the possible target gene of miR-26a, we designed luciferase reporters that included either the wild-type or mutant 3′-UTR of *SMAD1*. The dual-luciferase reporters were co-transfected with a miR-26a mimic or negative control (NC) into the DPCs, and miR-26a significantly reduced the firefly luciferase activity of the wild-type *SMAD1* reporter compared with the NC and no reduced activity was observed with the mutant luciferase reporter (*p* < 0.05 (Figure 10A)). The above results indicate that the seed region of miR-26a binds to a target-specific site in *SMAD1*. The results of western blotting showed that the miR-26a mimic inhibited the expression of the *SMAD1* protein (Figure 10B). Together, these results suggest that the protein expression of *SMAD1* were directly inhibited by miR-26a.

## 3. Discussion

Recent studies have demonstrated that miRNAs are widely involved in the occurrence and periodic growth of hair follicles [22,23,24,25]. These miRNAs, numerous regulatory factors and signal pathways constitute an extremely complex network regulation system in various types of hair follicle cells. Furthermore, the regulation of hair follicle morphogenesis and periodic development is achieved [26]. The shape, type and color of hair are determined not just during embryogenesis, but also repeatedly during each hair growth cycle [27]. To screen the miRNAs involved in the regulation of hair growth and the formation of striation patterns, a high-throughput sequencing of skin tissue from Zhongwei goats at 45 days and 108 days old was performed. We observed 28 differentially expressed miRNAs in the goat skin at the two developmental stages and verified 12 of these miRNAs by RT-qPCR. These miRNAs may be important factors influencing the changes in the hair bending phenotype. Some of them have been found to be related to the regulation of hair follicle development in previous reports. Zhang et al. identified multiple miRNAs in duck skin, including miR-10a and miR-451, which may regulate genes involved in the *Wnt/β-catenin*, *Shh/BMP* and *Notch* signal pathways [28]. Gao et al. detected 14 miRNAs participating in the regulation of the development of wool, including miR-143, miR-10a and let-7, among which miR-10a was differentially expressed between the large corrugated and small corrugated skins of Hu sheep [29]. Wen et al. screened 159 miRNAs expressed in skin and ear tissues, of which 105 were strongly conserved, and the miR-30 family was highly expressed in adult goat and sheep [30]. In our study, we detected some novel, significantly differentially expressed miRNAs that were not previously reported to be associated with hair bending or hair follicle development. From the RT-qPCR results, it can be seen that the difference between miR-130a and miR-26a is extremely significant compared to other miRNAs. Therefore, we chose miR-130 and miR-26a to further examine their function and effects on the DPC proliferation. The latest study found that miR-130a expression was increased in hypertrophic scar tissue and derived primary fibroblasts, which was accompanied by the upregulation of collagen 1/3 and *α-SMA* expression. The *miR-130a/CYLD/Akt* pathway may serve as a novel entry point for future skin fibrosis research [31]. Icli suggested that inhibiting miR-26a increased the mRNA level of its target gene *SMAD1* in the ECs at nine days post-wounding in diabetic mice. In addition, high glucose levels reduced the activity of the *SMAD1* 3′-UTR. Diabetic dermal wounds treated with LNA-anti-miR-26a had an increased expression of *ID1*, a downstream modulator of *SMAD1*, and a decreased expression of the cell cycle inhibitor p27 [32]. So far, great progress has been made on understanding the molecular mechanism underlying hair follicle regulation in human and model animals like mice and rats. The highly conserved characteristics of miRNAs are greatly convenient for understanding the relevant molecular mechanisms in animals.

MicroRNAs mainly exert their biological functions through interactions with target genes. In vivo, different gene products coordinate with each other to perform biological functions. An annotation analysis of the pathways for target genes of differentially expressed miRNAs can help further interpret the functions of miRNA. In the present study, pathway annotations indicate that DE miRNAs’ target genes are significantly enriched in the *TGF-β/SMAD* [33,34,35], *PI3K-Akt* [36], *JAK−STAT* [37], and *MAPK* [38], which are known to be involved in regulating hair follicle development, and miR-130a and miR-26a were among our key DE miRNAs of interest. Given the dramatic expression in the two development stages, miR-130 and miR-26a are selected as candidate miRNAs. They were predicted to have 1211 target genes and 1135 target genes, respectively. To further investigate the regulatory relationship between miRNAs and mRNAs in the hair development process, we integrated a miRNA-seq and transcriptional data from our laboratory. A miRNA target gene regulatory network associated with hair bending was constructed. Notably, the target genes of both miR-130a and miR-26a were enriched in the *TGF-β/SMAD* signal pathway. Studies have found that *TGF-β* plays an important regulatory role in cell growth and differentiation as well as hair follicle development and formation. *SMADs* are known as the special signal factors in the *TGF-β* signal pathway. When the corresponding receptor on the surface of the cell membrane binds to *TGF-β*, *SMADs* are responsible for transmitting the *TGF* signal from the receptor to the nucleus [39]. Previous studies have demonstrated that *SMADs* can regulate the development, periodic growth and pigmentation of hair follicles [40,41]. SMAD proteins can be roughly divided into three classes: receptor-activated SMADs, co-type SMADs and inhibitory SMADs. The receptor-activated SMADs (r-SMAD) include *SMAD1*, *SMAD5*, and *SMAD8*, which can specifically bind to the BMP protein, and *SMAD2* and *SMAD3*, which are the active substrates of the T-R1 receptor. The co-type SMADs (Co-SMAD), SMAD4, act as a co-required protein in the *TGF-1* pathway. The inhibitory SMADs (I-SMAD) include *SMAD6* and *SMAD7*, which inhibit *SMAD* phosphorylation and prevent signal transduction to the nucleus [42]. The regulatory networks of these two miRNA assemblies may contribute to the study of the mechanism of hair bending in Zhongwei goats.

Dermal papilla cells are mesenchymal cells that not only regulate the growth and development of hair follicles, but are also considered as a reservoir of pluripotent stem cells [43]. To determine the roles of miRNAs in regulating hair follicle development, we successfully isolated DPCs in vitro and provided a model to explore the miRNA potential functions. Dermal papilla cells are a group of cells located at the base of hair follicles that differentiate from dermal stromal cells, which have key regulatory effects on the morphogenesis and differentiation direction of hair follicles. Since Jahoda et al. [44] successfully isolated and cultured rat tentacled nipple cells, in vitro isolation and culture techniques for dermal papilla cells from other mammals (such as humans, pigs, sheep, and rabbits) have also been established [45,46,47,48]. Alpha-SMA is a specific marker of hair dermal papilla cells cultured in vitro, which is mainly used to distinguish these cells from fibroblasts [49]. VIM is a dermal hair follicle-derived cell marker for the identification of dermal-derived cells and epidermal-derived cells [50]. In this study, the expression of α-SMA and VIM was detected by immunofluorescence staining and western blotting of isolated cultured cells, indicating the successful isolation and acquisition of goat hair dermal papilla cells. This isolation of DPCs lays a foundation for subsequent gene function verification at the cellular level.

There are numerous reports on miR-130 and miR-26a in cancer and tumorigenesis, but research on hair follicles is still lacking. We first detected the effects of miR-130a and miR-26a on the proliferation of dermal papilla cells. The results showed that miR-130a could inhibit the proliferation of sheep dermal papilla cells, while miR-26a could promote the proliferation of these cells. Additionally, miR-130a has been shown to promote collagen secretion, myofibroblast transformation and cell proliferation by targeting CYLD and enhancing Akt activity [31]. It can be speculated that the same specific miRNA has different effects on different hair follicle cells. This may be due to differences in the periods of hair follicles, or it might be that the miRNA can play different roles in the regulation of hair follicles in different species. Next, we detected the changes in the expression levels of several *SMAD* genes in the *TGF-β/SMAD* pathway, which provided a molecular basis for further study on the occurrence and developmental mechanism of goat hair follicles. As expected, the results suggested that miR-130a and miR-26a can regulate the mRNA and protein expression level of the *SMAD* family. These miRNAs regulate the growth and development of hair follicles. Research has shown that the inhibition of miR-26a promotes angiogenesis and dermal wound healing in mice by increasing the endothelial *SMAD1* expression [32]. Our bioinformatic predictions suggested that *SMAD1* might be a target gene of miR-26a. Notably, the mRNA level of *SMAD1* does not change after the overexpression or inhibition of miR-26a. We further confirmed that miR-26a could reduce the protein expression of *SMAD1*. Therefore, we conclude that miR-26 plays a role after transcription.

## 4. Materials and Methods

### 4.1. Animals and Samples

All animal experimental procedures were approved by the Ministry of Agriculture of the People’s Republic of China and Institute of Animal Science, Chinese Academy of Agricultural Sciences and were performed according to the guidelines for the care and use of experimental animals established by this ministry. Before collecting samples, we obtained the permission of Ningxia Zhongwei Goat Conservation Farm. Three Zhongwei goats located at a Zhongwei goat breeding farm in Ningxia, China, were randomly selected and had no relationship with each. We collected skin tissue from the goats at two time points: specifically, three 1 cm^2^ pieces of skin tissue were collected from the scapula using a sterilized scalpel. Some samples were immediately stored in RNAlater (Thermo Fisher Scientific, NY, USA) and kept at −80 °C until further processing. Other samples were quickly stored in a 4% paraformaldehyde fixative to prepare the paraffin sections. All wounds were treated with Yunnan Baiyao Powder to stop bleeding (China Yunnan Baiyao Group Co., Ltd., Kunming, China). 

Ethical approval for animal survival was provided by the animal ethics committee of the Institute of Animal Science, Chinese Academy of Agricultural Sciences (IAS-CAAS) with the following reference number: IASCAAS-AE-03, on 1 September 2014.

### 4.2. Total RNA Extraction and Small RNA Library Preparation

Total RNA was extracted according to the RNeasy Plus Universal Mini Kit method. To ensure the accuracy of the data obtained, a nanodrop was used to detect whether the purity, concentration, and nucleic acid absorption peak of the isolated RNA were normal, and an Agilent 2100 was used to accurately evaluate the RNA integrity. Starting with the total RNA, a connector was added to each end of small RNA transcripts, and cDNA was synthesized by reverse transcription. Subsequently, after PCR amplification, we separated the target DNA fragments by PAGE. A cDNA library was obtained by gelatinization recovery.

### 4.3. Small RNA Sequencing and Read Processing

The libraries were sequenced on an Illumina HiSeq 2500 platform, and 50 bp single-end reads were generated. We conducted a series of data quality assessments, including an inspection of error rates, removal of low-quality reads, and length selection. Next, we analyzed the length distribution statistics. The peak of the length distribution helps determine the small RNA species. We used Bowtie [51] to compare the screened reads to a reference sequence (ftp://ftp.ncbi.nlm.nih.gov/genomes/refseq/vertebrate_mammalian/Capra_hircus/latest_assembly_versions/GCF_001704415.1_ARS1) and analyzed the distribution of the reads along the reference genome. To remove the tags that corresponded to low-complexity sequences (including rRNA, tRNA, snRNA and snoRNA), we compared the clean reads with the GtRNAdb database [52], Rfam database [53], Repbase database [54] and NCBI database [55]. Candidate miRNAs were obtained after deduplication. To identify miRNAs conserved among species, we compared the reads obtained by sequencing with those in other mammalian miRBase 22.0 databases (1 to 2 base mismatches were allowed during the process). The expression of the known and new miRNAs in each sample were counted, and the expressions were normalized with *TPM*.

TPM=Readcount×1,000,000Mapped Reads

A differential expression analysis of two groups of data was performed using DESeq [56]. The differential miRNA screening conditions were FDR < 0.01 and |fold change| > 1.5.

### 4.4. The Sequencing Results for Small RNA Were Verified by RT-qPCR

Differentially expressed miRNAs were randomly selected for RT-qPCR. The U6 gene was used as a reference, and primers were designed and synthesized by Shanghai Bioengineering Co., Ltd. (Shanghai, China). The total RNA isolated from the above-described 6 samples (3 from each 45-day-old and 108-day-old lamb) was subjected to reverse transcription cDNA synthesis. Reverse transcription cDNA synthesis was carried out in accordance with the Takara reverse transcription kit instructions. The products were stored at −20 °C until use. The reverse transcription products were used as templates for quantitative RT-qPCR. RT-qPCR was performed with reference to Takara real-time quantitative kit specifications: reaction system (20 μL); cDNA (2 μL), upstream and downstream primers (0.8 μL (Table S1)), SYBR (10 μL), ddH_2_O (6.4 μL). The test results were converted into relative expression data using the 2^−ΔΔCt^ method for relative quantitative analysis. A *t* test was performed, and *p* < 0.05 was defined as a significant difference. The reaction system was placed on an ABI quantitative PCR instrument for reaction. At least three samples were included for each time point, and all reactions for each sample were repeated three times.

### 4.5. Target Gene Prediction and Functional Notation of Differentially Expressed miRNAs

To better understand the biological functions of the identified miRNAs, we used RNAhybrid [57] and miRanda [58] software to predict the miRNA target genes. Gene Ontology (GO; http://www.geneontology.org/) is an international standard classification system for gene function. The Kyoto Encyclopedia of Genes and Genomes (KEGG; https://www.kegg.jp/kegg/) is a system for analyzing gene function and genomic information databases. We screened target genes of differentially expressed miRNAs for experimental purposes, and enrichments in the distribution of the target genes were assessed by a GO analysis. Additionally, the gene-enriched pathways of the target genes of the differentially expressed miRNAs were detected by a KEGG analysis to elucidate the expression pattern in each sample in the experiment by evaluating the functions of the target genes.

### 4.6. Differentially Expressed miRNA-Target Gene Interaction Network Analysis

Prior to a differential gene expression analysis for each sequenced library, a differential expression analysis of two samples was performed using IDEG6. The p value was adjusted using the q value. An FDR < 0.01 and |foldchange| ≥ 1.5 were set as the threshold for significant differential expression. MicroRNAs associated with the research purposes were selected from validated differentially expressed miRNAs. The predicted miRNA target genes were combined with transcriptomic analysis data to construct an mRNA−miRNA network. Transcriptome analysis data have been published, and the NCBI accession number is PRJNA555706. The software Cytoscape 3.4.0 was used to graphically visualize the network [59].

### 4.7. Dermal Papilla Cell Separation and Culture

Approximately 1 cm^2^ of scapular skin tissue was removed by aseptic surgery, placed in DMEM containing two antibodies, and stored in an ice box. The harvested skin tissue was immersed in a petri dish containing 75% alcohol for 1 min and was rapidly washed 4–5 times with PBS. The tissue pieces were cut into 1 mm^2^ pieces using a sterile surgical blade, and these small tissue pieces were then collected in a petri dish containing 0.25% neutral protease and were incubated at 37 °C for approximately 2 h. Under a dissecting microscope, the dermal layer and subcutaneous tissue layer were gently separated with high-precision forceps to expose hair follicle bulges. The bulges were collected in a 1.5 mL centrifuge tube (including 1 mL of complete medium). We centrifuged the tube at 1500 rpm for 5 min and discarded the waste; the pellet in the tube was the size of a soybean. Then, 400 µL of type IV collagenase was added, and the precipitate was thoroughly mixed. After 15 min, the digestion was terminated, and the mixture was centrifuged. The cell mixture was passed through a 150 µm mesh cell sieve and seeded in a cell culture flask for cultivation.

### 4.8. Dermal Papilla Cell Identification

Fourth-passage cells in good growth conditions were seeded in petri dishes and cultured in an incubator at 37 °C for 48 h. After the cell density reached 70~80%, the cultivation was terminated. The expression of α-smooth muscle actin (*α-SMA*) and vimentin (*VIM*), a specific marker of hair papilla cells cultured in vitro, were detected by immunofluorescence staining. The total protein was extracted from hair dermal papilla cells in vitro, and the expression of specific markers was detected by Western blotting.

### 4.9. Overexpression and Inhibition of miR-26a and miR-130a

miRNA mimics and inhibitors were formulated in 20 µM stock solutions for transfection experiments according to miRNA product instructions (Shanghai Jiama Biotechnology Co., Ltd., Shanghai, China). The mimic and inhibitor sequences are showed in Table 2. Cells were transfected with miR-26a/miR-130a mimics or inhibitors according to the instructions for Lipofectamine 3000 (Invitrogen, Carlsbad, CA, USA). The transfection groups are as follows: miR-26a mimic/miR-26a mimic-NC, miR-26a inhibitor/miR-26a inhibitor-NC, miR-130a mimic/miR-130a mimic-NC, miR-130a inhibitor/miR-130a inhibitor-NC. We extracted RNA from hair papillary cells, and RT-qPCR was performed to detect the overexpression and inhibition effect. Methods and reagents are shown in Part 4.4 of this section. The primers for RT-qPCR are shown in Table S2.

### 4.10. Cell Proliferation Assay

Forty-eight hours after transfection, dermal papilla cells were digested with 0.25% trypsin. After 5 min, the digestion was terminated, and the medium was centrifuged. The medium was added to a cell suspension, which was seeded in a 96-well plate at 100 µL/well (approximately 1 × 104 cells) and cultured in an incubator at 37 °C in saturated humidity and 5% CO_2_. The experiment included a control group (no treatment) and an experimental group, and each group comprised 6 parallel wells. The old medium was discarded after 0, 12, 24, 36, or 48 h of incubation, and the cells were washed twice with PBS. Next, we added 10 µL of CCK-8 solution to each well (avoiding the generation of air bubbles) and continued to incubate the plate for 2 h in a 37 °C incubator. Finally, the OD values were measured at 450 nm by an enzyme-labelling instrument. The relative proliferation level of hair dermal papilla cells is represented by the OD value.

### 4.11. RNA Extraction, Reverse Transcription and RT-qPCR

Three days after the dermal papilla cells were transfected, a total RNA extraction was performed using the miRNeasy Mini Kit (QIAGEN, Dusseldorf, Germany). Reverse transcription cDNA synthesis was carried out in accordance with the Takara reverse transcription kit instructions. A quantitative analysis was performed using SYBR Premix Ex TaqTM II (Taiwan TaKaRa Bio., Taibei, China) and an ABI quantitative PCR instrument. The primers used for the analysis are listed in the attached table. The experimental results were analyzed by the 2^−ΔΔCt^ method. A t test was performed, and *p* < 0.05 was defined as a significant difference. Three samples were included for each time point, and all the reactions for each sample were repeated three times. The primers for RT-qPCR are shown in Table S3.

### 4.12. Western Blot Analysis

Based on previous data analysis and experimental results, we selected *SMAD2* and *SMAD6* in the *TGF-β/SMAD* signal pathway for protein level validation. We extracted the total protein from dermal papilla cells 3 d after transfection. Western blot analysis mainly included the following steps: preparation of a colloid, transfer to a membrane, blocking, binding of a specific antibody to the corresponding target protein, and visualization. The primary antibodies used were murine anti-β-actin (1:1000 (Abcam, Cambridge, UK)), murine anti-SMAD2 (1:1000 (Abcam, Cambridge, UK)), and rabbit anti-SMAD6 (1:1000 (Abcam, Cambridge, UK)). The antibody was diluted in a 5% BSA blocking solution (Beijing Solable Technology Co., Ltd., Beijing, China).

### 4.13. Luciferase Reporter Gene Assay

According to the dual-luciferase reporter product instructions, (Shanghai Jiama Biotechnology Co., Ltd.), the recombinant mutant vector SMAD1-miR26a-MUT and the wild-type vector SMAD1-miR26a-WT were cotransfected with the miRNA mimic miR-26a mimetic or miR-26a mimic-NC. Forty-eight hours after cotransfection, the activities of the Renilla luciferase and firefly luciferase were measured by a dual-luciferase reporter assay kit (Promega, Madison, WI, USA). At least 3 samples were included for each time point, and all reactions for each sample were repeated three times.

### 4.14. Statistical Analysis

All data are presented as the mean ± S.D. based on at least three replicates for each treatment. The data were analyzed by performing one-sample *t* tests.

### 4.15. Availability of Data and Materials

This manuscript contains previously published data. The sample information for transcriptome sequencing data were submitted to the NCBI BioProject section under accession number PRJNA555706.

## 5. Conclusions

In summary, we characterized the miRNA expression profiles of Zhongwei goat skins in different developmental stages with diverse wool curvature patterns, and detected a set of miRNAs and target genes related to hair follicle development, and constructed a miRNA−mRNA regulatory network. In addition, we also identified some pathways associated with hair follicle development, including the *TGF-β/SMAD*, *PI3K-Akt*, *JAK-STAT*, and *MAPK* pathways. This study also contributed to the annotation of the goat genome. The study revealed that miR-26a and miR-130a were associated with the growth and development of hair follicles, which affected the proliferation of dermal papilla cells. In addition, our findings confirmed that the *TGF-β/SMAD* signal pathway was involved in the development of hair follicles. Our research provided a theoretical basis for further screening of molecular markers related to hair bending in goats.

## Figures and Tables

**Table 1:** Statistics of differentially expressed microRNAs (miRNAs).

ID	D108_Count	D45_Count	D108_TPM	D45_TPM	FDR	log2FC
chi-let-7a-5p	52	7	9.8407	1.4161	1.61 × 10^−05^	−3.0405
chi-miR-451-5p	55	9	10.4084	1.8207	4.72 × 10^−05^	−2.7954
chi-miR-26a-5p	162	11	24.4730	4.5195	8.50 × 10^−03^	−2.7095
chi-miR-10a-5p	40	7	7.5697	1.4161	7.96 × 10^−04^	−2.6681
chi-miR-1468-5p	31	6	5.8666	1.2138	7.28 × 10^−03^	−2.5028
chi-miR-204-5p	122	27	23.0877	5.4620	5.33 × 10^−06^	−2.4410
chi-miR-30a-3p	640	146	121.1160	29.5350	1.56 × 10^−08^	−2.4293
chi-miR-9-3p	72	16	13.6255	3.2367	1.26 × 10^−04^	−2.4089
chi-miR-197-3p	45	12	8.5160	2.4275	8.07 × 10^−03^	−2.1288
chi-miR-144-3p	167	50	31.6037	10.1147	1.06 × 10^−04^	−2.0246
chi-miR-187-5p	28	108	5.7319	38.6300	1.18 × 10^−06^	2.4468
chi-miR-424-5p	232	576	47.4927	206.0269	1.24 × 10^−05^	1.8399
chi-miR-100-5p	16	46	3.2754	16.4535	5.98 × 10^−03^	2.0036
chi-miR-542-5p	17	50	3.4801	17.8843	3.25 × 10^−03^	2.0390
chi-miR-301a-3p	22	71	4.5036	25.3957	1.85 × 10^−04^	2.1827
unconservative_NW_017189517.1_39211	1514	5051	309.9306	1806.6695	1.77 × 10^−09^	2.2690
chi-miR-2332	2	16	0.3021	6.5738	3.35 × 10^−03^	3.5404
chi-miR-215-5p	124	508	23.4662	102.7657	1.18 × 10^−03^	1.7239
chi-miR-10b-5p	13	55	1.9639	22.5973	8.80 × 10^−05^	3.0961
chi-miR-16a-5p	7	32	1.4330	11.4459	1.40 × 10^−03^	2.5969
chi-miR-17-3p	7	33	1.4330	11.8036	9.25 × 10^−04^	2.6405
chi-miR-3432-5p	7	68	1.3247	13.7560	1.03 × 10^−04^	2.8494
chi-miR-128-3p	8	81	1.5140	16.3859	2.06 × 10^−05^	2.9235
unconservative_Chr1_79	25	100	5.1177	35.7686	1.19 × 10^−06^	2.4952
chi-miR-19a	6	75	1.1355	15.1721	7.83 × 10^−06^	3.1885
chi-miR-130-5p	340	1195	69.6013	427.4342	7.22 × 10^−10^	2.3422
chi-miR-18a-3p	6	86	1.2283	30.7610	1.07 × 10^−12^	4.2068
chi-miR-2284b	4	44	0.6043	18.0778	5.27 × 10^−07^	4.2491

**Table 2:** The mimics and inhibitors sequences.

MicroRNA Name	Sense (5′–3′)
miR-26a	UUCAAGUAAUCCAGGAUAGGCU
miR-130a	GCUCUUUUCACAUUGUGCUACU
microRNA mimics NC	UUGUACUACACAAAAGUACUG
mircoRNA inhibitor NC	CAGUACUUUUGUGUAGUACAA

## Supplementary Materials

Supplementary materials can be found at https://www.mdpi.com/1422-0067/21/14/5076/s1.

## Abbreviations

miRNA	microRNA
DE	different expression
Seq	sequence
GO	Gene Ontology
KEGG	Kyoto Encyclopedia of Genes and Genomes
PAGE	polyacrylamide gel electrophoresis
RT-qPCR	Quantitative real time polymerase chain reaction
